# Isolation, Characterization, Moisturization and Anti-HepG2 Cell Activities of a Novel Polysaccharide from *Cyanobacterium aponinum*

**DOI:** 10.3390/molecules29194556

**Published:** 2024-09-25

**Authors:** Zishuo Chen, Jiayi Wu, Na Wang, Tao Li, Houbo Wu, Hualian Wu, Wenzhou Xiang

**Affiliations:** 1 CAS Key Laboratory of Tropical Marine Bio-Resources and Ecology, Guangdong Key Laboratory of Marine Materia Medica, Institution of South China Sea Ecology and Environmental Engineering, RNAM Center for Marine Microbiology, South China Sea Institute of Oceanology, Chinese Academy of Sciences, Guangzhou 510301, China; zschen1030@gmail.com (Z.C.); wujiayi@ipm-gba.org.cn (J.W.); wangna@usc.edu.cn (N.W.); taoli@scsio.ac.cn (T.L.); wuhoubo@scsio.ac.cn (H.W.); 2 University of Chinese Academy of Sciences, Beijing 100049, China; 3 Greater Bay Area Institute of Precision Medicine (Guangzhou), Guangzhou 511466, China; 4 School of Basic Medical Sciences, Heyang Medical School, University of South China, Hengyang 421001, China

**Keywords:** *Cyanobacterium aponinum* SCSIO-45682, water-soluble polysaccharides, characterization, moisture retention activity, cytotoxicity against HepG2 cells

## Abstract

Polysaccharides from cyanobacteria are extensively reported for their complex structures, good biocompatibility, and diverse bioactivities, but only a few cyanobacterial species have been exploited for the biotechnological production of polysaccharides. According to our previous study, the newly isolated marine cyanobacterium *Cyanobacterium aponinum* SCSIO-45682 was a good candidate for polysaccharide production. This work provided a systematic study of the extraction optimization, isolation, structural characterization, and bioactivity evaluation of polysaccharides from *C. aponinum* SCSIO-45682. Results showed that the crude polysaccharide yield of *C. aponinum* reached 17.02% by hot water extraction. The crude polysaccharides showed a porous and fibrous structure, as well as good moisture absorption and retention capacities comparable to that of sodium alginate. A homogeneous polysaccharide (*Cyanobacterium aponinum* polysaccharide, CAP) was obtained after cellulose DEAE-52 column and Sephadex G-100 column purification. CAP possessed a high molecular weight of 4596.64 kDa. It was mainly composed of fucose, galactose, and galacturonic acid, with a molar ratio of 15.27:11.39:8.64. The uronic acid content and sulfate content of CAP was 12.96% and 18.06%, respectively. Furthermore, CAP showed an in vitro growth inhibition effect on human hepatocellular carcinoma (HepG2) cells. The above results indicated the potential of polysaccharides from the marine cyanobacterium *C. aponinum* SCSIO-45682 as a moisturizer and anticancer addictive applied in cosmetical and pharmaceutical industries.

## 1. Introduction

Cyanobacteria, previously known as blue-green algae, are Gram-negative prokaryotes capable of oxygenic photosynthesis. They are fundamental contributors to multiple biogeochemical cycles [1], with marine cyanobacteria being responsible for 25% of net primary productivity in the ocean [2,3]. Cyanobacteria are ubiquitously present in every habitat or almost in extreme environments, such as exposure to Gamma or UV radiation, heat, salinity, and heavy metals [4,5,6]. In recent decades, this primitive and unique phylum has attracted much attention from both academia and industry due to its simple growth requirements, ease of genetic manipulation, and sustainable synthesis of various bioactive compounds [7]. Among the high-value products derived from cyanobacteria, polysaccharides have recently been highlighted. Cyanobacteria own a complex carbohydrate metabolic mechanism about the synthesis of intracellular and extracellular polysaccharides serving as a reserve (glucans), structural cell components (pectin and cellulose), and a protector for the cell from the adverse environment [8,9,10,11]. Moreover, some cyanobacterial polysaccharides exhibit diverse biological activities, including antiviral, antioxidant, anti-inflammatory, antimicrobial, antitumor, and moisture retention capacities, which endue them with potential applications in food, agriculture, pharmaceutical, and cosmetic fields [6,11,12,13].

Moisturizing property is one of the remarkable bioactivities shown by cyanobacterial polysaccharides. Studies on *Nostoc commune* showed that polysaccharides from *N. commune* exhibited notable moisture absorption and retention capabilities, compared with urea and chitosan, as well as good water retention efficacy in mouse stratum corneum [14]. Moreover, Sacran, a massive jelly-like anionic exopolysaccharide excreted by *Aphanothece sacrum*, demonstrates outstanding moisture retention and skin barrier functions applicable in a wide range of skincare products [15]. Sacran consists of 11 types of monosaccharides, 12% carboxyl groups per sugar chain, and approximately 11% sulfate groups [16,17]. Such extracellular polymeric substances are hydrated macromolecules that contain sulfate groups, neutral sugar (such as glucose, galactose, mannose, fructose, ribose, xylose, arabinose, fucose and rhamnose) residues, non-carbohydrate constituents such as phosphate, lactate, acetate and glycerol, as well as uronic acid (glucuronic and galacturonic acid). Their well-known moisture retention capacity is attributed to the strong interactions between water molecules and the hydrophilic -OH groups of the polysaccharides [15]. In general, the water-holding ability is closely related to molecular weight, functional groups, polysaccharide modifications, and apparent structure of polysaccharides [18]. Polysaccharides exhibit moisture retention activity by hydrogen bonding with water molecules, formation of a three-dimensional network structure to prevent water escape, and generation of hydration films to avoid water evaporation. Some polysaccharides also interact with skin intrinsically by regulating the synthesis of tight junction proteins, barrier proteins, water channel proteins, etc. [18]. One of the most common polysaccharides applied in cosmetics is hyaluronic acid, a non-sulfated glycosaminoglycan composed of repeating polymeric disaccharides [19]. However, the limited sources of hyaluronic acid, which are mainly connective tissues from animals, lead to a high production cost [20]. Thus, it is important to screen for polysaccharides from non-animal origin. Cyanobacteria stand out as microscopic autotrophs for massive production without competing for arable land [7,21]. *Anabaena* spp., *Calothrix marchica*, *Cyanospira capsulate*, *Cyanothece* sp., *Leptolyngbya* sp. *Plectonema* sp., *Nostoc* spp., *Gloeocapsa gelatinosa*, *Oscillatoria simplicissima*, *Spirulina platensis*, etc., were reported to be potential polysaccharide producers [21,22,23].

Apart from displaying a moisturizing effect, cyanobacterial polysaccharides have emerged as natural antitumor agents. They could be developed as an alternative to existing cancer chemotherapy by possessing selective activity against tumor cells with minimal toxic side effects [24,25]. Mishima et al. reported that calcium spirulan, a sulfated polysaccharide derived from *Spirulina platensis*, could reduce the lung metastasis of B16-BL6 melanoma cells by inhibiting tumor invasion and metastasis [26]. Gacheva et al. [27] reported that exopolysaccharides from *Gloeocapsa* sp. significantly decreased the viability of human cervical carcinoma cells, HeLa. The prolonged cultivation period of *Gloeocapsa* sp. led to more than twice the increase in cytotoxicity of the derived exopolysaccharides. Guo et al. [28] reported that a polysaccharide from *N. commune* Vauch significantly suppressed the growth and proliferation of MCF-7 and DLD1 cells. This polysaccharide simultaneously triggered intrinsic, extrinsic, and endoplasmic reticulum stress-mediated apoptotic signaling pathways. Flores et al. [29] reported the antitumor activity of the extracellular carbohydrate polymer from *Synechocystis ΔsigF* overproducing mutant. They found that the *ΔsigF* polymer significantly decreased the viability of melanoma, thyroid, and ovary carcinoma cells by inducing high levels of apoptosis through p53 and caspase-3 activation. It is worth noting that other than polysaccharides, a diversity of bioactive components, e.g., C-phycocyanin, vitamins, terpenoids, phenolic acids, flavonoids, and alkaloids, isolated from cyanobacteria have shown promising anticancer properties, targeting a variety of cancer cells. This demonstrates the potential of cyanobacteria as a potent source of natural antitumor metabolites when more research is implemented [30,31,32].

The present research was focused on one newly isolated marine cyanobacterial strain named *Cyanobacterium aponinum* SCSIO-45682. According to our previous study, this strain had good adaptation to high alkalinity; when 8.4 g L^−1^ of sodium bicarbonate was supplied as the carbon source in a standing Erlenmeyer flask, this strain reached a high biomass yield of 2.48 g L^−1^ and a high total carbohydrate level of 44.92% of dry weight (DW) [33]. *C. aponinum*, belonging to Chroococcales, was first isolated in 2007 by Moro et al. [34] from cyanobacterial mats in the Euganean thermal springs of Italy. This species is characterized by a simple spherical morphology and reproduction via binary fission. *C. aponinum* is widely distributed in nature and has been isolated from freshwater hot springs [34,35,36], marine hot springs [37], reed beds [38], and even deserts [39]. It is a polyextremophile capable of tolerating extreme conditions such as high temperature (50 °C), acidic stress (pH 3.0), high CO_2_ concentrations (10%, *v:v*) [37], salt stress (70 g L^−1^ NaCl) [38], and high light intensity (3200 µmol photon m^−2^ s^−1^) [39], wastewater [40] and geothermal gas [41]. *C. aponinum* also demonstrates great potential for the accumulation of versatile high-value compounds, e.g., exopolysaccharides [42], C-phycocyanin [40,43,44], zeaxanthin, *β*-carotene [42] as well as lipids [45], glycogen and protein [46]. The extracellular polysaccharides secreted from *C. aponinum* displayed significant immunomodulatory and anti-inflammatory effects, potentially beneficial for the treatments of psoriasis [47,48]. Due to its significant skincare properties, fermented products of *C. aponinum* have been included in the International Nomenclature of Cosmetic Ingredients list [49]. Hence, *C. aponinum* stands out as an emerging cyanobacterial resource with a high biomass yield, robust adaptability to extreme environments, and the ability to accumulate different value-added metabolites. However, as far as we know, the water-soluble polysaccharides from marine *C. aponinum* have not been reported yet.

Thus, we conducted a systematic study about extraction optimization, isolation characterization, moisture retention, and anti-liver cancer activities investigation of water-soluble polysaccharides from *C. aponinum* SCSIO-45682. These results may provide a theoretical basis for the exploration of *C. aponinum* polysaccharides as moisturizers and a possible source of antitumor agents, as well as contribute to the application of cyanobacterial polysaccharides in functional food, pharmaceuticals, and cosmetics.

## 2. Results and Discussion

### 2.1. Extraction Optimization of Crude Polysaccharides from C. aponinum SCSIO-45682

The selection of a suitable extraction method is a crucial step as it may affect the yield, composition, structure, and integrity of polysaccharides [50]. There are various approaches available for the extraction of polysaccharides, including hot water, dilute acid, dilute alkali, and microwave, ultrasonic, and enzyme-assisted methods [51]. In general, hot water extraction is the most common method for extracting polysaccharides, and its advantages include no requirement for special equipment, low cost, easy to operate, and suitable for large-scale industrial use [52,53]. Additionally, it is suitable for the preparation of neutral and acidic polysaccharides [54], in contrary to the degradation caused by diluted alkali or diluted acid extraction [55]. The extraction yield through this method largely depends on a liquid-to-solid ratio, extraction temperature, extraction time, and extraction times [56]. Orthogonal test design is a common approach for extraction optimization, which can establish a small number of testing schemes to investigate the influences of multiple factors and multiple levels on a certain result. Based on the mathematical analysis, the optimal combined condition in the extraction process will be generated [57].

According to Figure S1, a preliminary single-factor experiment about the effects of four independent variables, i.e., solid-liquid ratio (A), extraction temperature (B), extraction time (C), and extraction times (D), on the water-soluble crude polysaccharide yield was carried out. The highest extraction yield reached 10.73%, 11.98%, 11.88%, and 15.79% DW, respectively, when a solid-liquid ratio of 1:40 (g: mL), an extraction temperature of 70 °C, extraction time of 2 h, and extraction times of 5 were adopted, respectively. Thus, solid-liquid ratios of 1:30, 1:40, and 1:50, extraction temperatures of 60 °C, 70 °C, and 80 °C, extraction periods of 1 h, 2 h, and 3 h, and extraction times of 3, 4, and 5 were chosen for the following orthogonal assay.

As presented in Table 1, an orthogonal assay with four factors at three levels L_9_ (3^4^) was conducted to optimize the crude polysaccharides yield from *C. aponinum* SCSIO-45682. The results showed that the third run (A_1_B_3_C_3_D_3_, under a solid-liquid ratio of 1:30, an extraction temperature of 80 °C, and an extraction period of 3 h for 5 times) obtained the maximum yield of 17.02% DW. Based on R values, the four parameters had an influence on the polysaccharide yield in the following order: C > D >A > B. According to the results of the corresponding variance analysis (Table 2), if factor B (minimum error) was regarded as a random error, factors C and D had a significant impact on the polysaccharide extraction efficacy (*p* < 0.05). The A_1_B_3_C_3_D_3_ combination would, therefore, be the most effective hot water extraction condition. Additionally, it was noted that when 8.4 g L^−1^ sodium bicarbonate was supplied as the carbon source (Section 3.1), the total carbohydrate, crude protein, and total lipid constituted 44.92%, 25.68%, and 17.53% DW, respectively, of *C. aponinum* SCSIO-45682 biomass [33]. This showed a high extraction rate of carbohydrate polymers in our present study.

### 2.2. Morphological Characterization of Crude Polysaccharides from C. aponinum SCSIO-45682 

**Figure 1:**
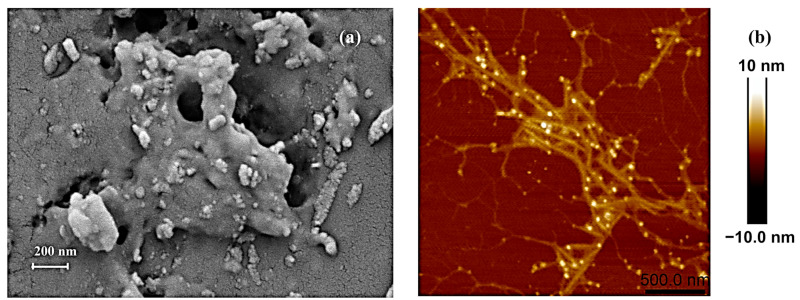
Morphological features of crude water-soluble polysaccharides from *C. aponinum* SCSIO-45682 under scanning electron microscopy (×50,000) (**a**) and atomic force microscopy (scale bar: 500 nm) (**b**).

After hot water extraction and partial purification to remove free proteins, pigments, and small molecules, the water-soluble crude polysaccharides obtained were subjected to morphological observation. Scanning electron microscopy (SEM) was employed to monitor the porosity, size, and shape features of the crude polysaccharides. As shown in Figure 1a, it exhibited a porous appearance with a relatively rough surface (under 50,000× magnification). Notably, in polysaccharides and their derivatives, the porous structure may have a positive correlation with their water-holding and swelling properties [58]. Atomic force microscopy (AFM), as a high-resolution tool, was frequently used to observe the chain conformation and height of the polysaccharides. Figure 1b reveals visible aggregates of crude polysaccharides in fibrous form, largely branched, with the height significantly exceeding the average height (0.1–1.0 nm) of single-chain polysaccharide molecules. *C. aponinum* SCSIO-45682 polysaccharides featured a highly branched structure, and Wu et al. reported that this kind of structure may improve the viscosity and water-holding capacity of polysaccharides [59].

### 2.3. Isolation of Crude Polysaccharides from C. aponinum SCSIO-45682

**Figure 2:**
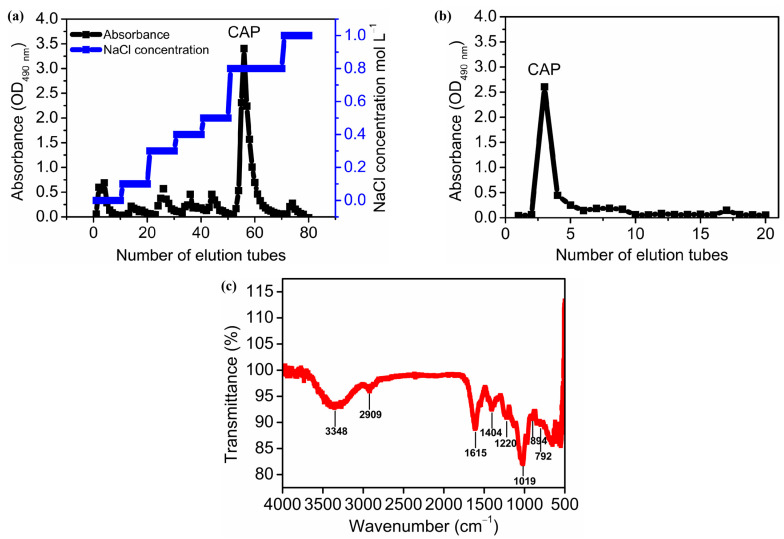
Isolation of CAP using ion exchange column chromatography (DEAE-cellulose-52 column eluted by water and a series of NaCl solutions (0.1, 0.3, 0.4, 0.5, 0.8, 1.0 M)) (**a**) and size-exclusion chromatography (Sephadex G-100 column eluted by water) (**b**) as well as Fourier-transform infrared spectrum of CAP (**c**). Note: (**a**) shows the sugar-specific absorbance of collected samples through the DEAE-cellulose-52 column, eluted by water and a series of NaCl solutions (0.1, 0.3, 0.4, 0.5, 0.8, 1.0 M). (**b**) shows the sugar-specific absorbance of collected samples through a Sephadex G-100 column eluted by water.

Figure 2a,b depicts the isolation and purification of polysaccharides using the column chromatography method. Crude polysaccharides were first applied to a DEAE-52 cellulose column, eluted with ultrapure water and sodium chloride solutions at a series of concentrations, resulting in 7 components (Figure 2a). The most predominant fraction was obtained by elution with 0.8 M NaCl. The recovery rate was 39.99% of the crude polysaccharides (*w:w*). Subsequently, this major fraction was applied to a Sephadex G-100 column, eluted with ultrapure water, and yielded a single, symmetrical peak with a recovery rate of 25.54% of the crude polysaccharides (Figure 2b). This major fraction was further characterized as one homogeneous polysaccharide and named *Cyanobacterium aponinum* polysaccharide (CAP).

### 2.4. Homogeneity and Molecular Weight of CAP

The homogeneity and molecular weight (Mw) distribution of CAP, measured by a high-performance size-exclusion chromatography-multi angle laser light scattering-refractive index detector (HPSEC-MALLS-RI), was represented as the profile of the multi-angle laser light scattering detector. The profile exhibited a single, symmetric peak, indicating the homogeneity of CAP (Figure S2). Calculations revealed an absolute Mw of 4596.64 kDa (Table 3) and a polydispersity index (Mw/Mn) of 12.82, which indicated that CAP was a relatively homogeneous, high molecular weight polymer. 

### 2.5. Monosaccharide Composition of CAP

As demonstrated in Table 3, CAP was composed of ten kinds of monosaccharide block units, i.e., fucose, galactose, galacturonic acid, glucose, rhamnose, ribose, mannose, guluronic acid, arabinose, and xylose in a molar ratio of 15.27:11.39:8.64:7.08:4.53:4.43:4.07:1.30:0.31:0.26. Interestingly, unlike polysaccharides from other cyanobacteria, CAP was remarkably rich in fucose. This shares some similarities with fucoidan produced by marine brown seaweeds *Phaeophyceae* sp. Fucoidan is a fucose-rich sulfated polysaccharide known for anticancer, immunoregulatory, anticoagulation, and antithrombotic activities [60]. The hot water extracted polysaccharides from another marine brown macroalga, *Sargassum thunbergia,* were also predominantly rich in fucose, followed by galactose, mannose, xylose, glucose, and glucuronic acid [55].

### 2.6. Chemical Composition of CAP

The chemical compositions of CAP are summarized in Table 3. The total carbohydrate content of CAP was 34.69%, comparable to the sulfated polysaccharides extracted from *Spirulina platensis* by NaOH (38.7%) [61]. The protein content of CAP was as low as 0.84%, showing a good deproteinization effect and negligible existence of polysaccharide-associated proteins. The total phenolic compound accounted for 0.42% of CAP, much lower than of polysaccharides from corn silk by fractional precipitation, which reached 7.71–10.30% [62]. Notably, the sulfate and uronic acid group levels reached 18.06% and 12.96%, respectively, showing an acidic polysaccharide nature of CAP. Research shows that the presence of sulfate and uronic acid groups in polysaccharides may be closely associated with their antioxidant, antiviral, anticancer, and immunoregulatory effects [63,64]. The total carbon content of CAP was 34.68%, which was consistent with the value proposed by Martin et al. that carbon accounts for 40% of polysaccharides [65].

### 2.7. FT-IR Analysis

FT-IR is an effective tool for determining characteristic functional groups in the backbone and branches of polysaccharides. Figure 2c shows the FT-IR spectrum of CAP between 4000 and 500 cm^−1^. The peak at 3448 cm^−1^ was attributed to the stretching vibration of O-H, whereas the peaks at 2909, 2844, and 1447 cm^−1^ resulted from the stretching vibration and bending vibration of C-H, indicating the typical absorption peaks of polysaccharides [54]. The peak at 1615 and 1404 cm^−1^ were referred to as the stretching vibration of C=O, indicating the presence of uronic acids [66], which was consistent with the finding that CAP contained relatively high amounts of uronic acids (12.96%) (Table 3). The band at 1220 cm^−1^ was due to the stretching vibration of O=S=O [67], indicating the presence of sulfate in CAP (Table 3). The strong absorption at 1019 cm ^−1^ was dominated by the stretching vibration of the pyranose ring, which confirmed the existence of pyranose in the CAP [68]. Moreover, a medium peak at 792 cm^−1^ and a weak peak at 894 cm^−1^ belonged to β- and α-glycosidic bonds in CAP [69].

### 2.8. Moisture Absorption and Retention Capacities of Crude Polysaccharides

**Figure 3:**
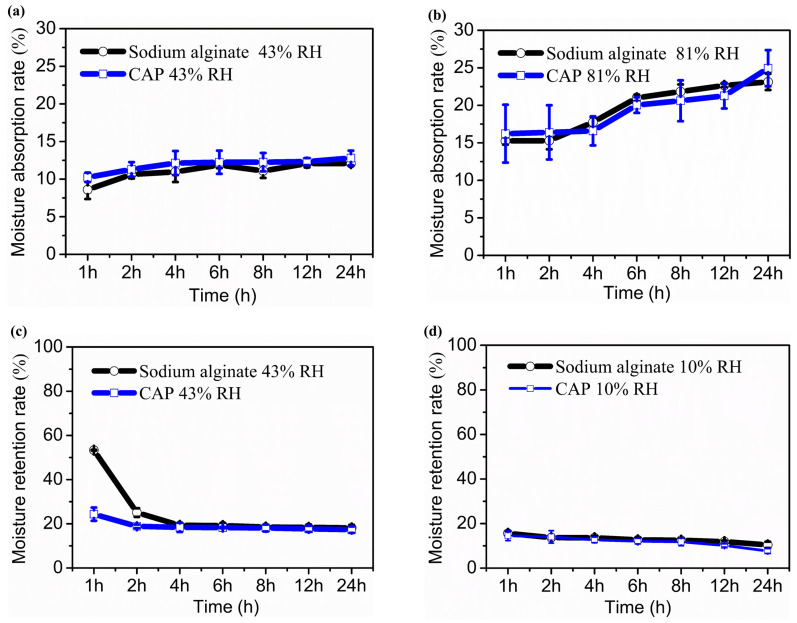
Moisture absorption and retention activities of crude polysaccharides from *C. aponinum* SCSIO-45682 in vitro. Moisture absorption rate under 43% RH (**a**), moisture absorption rate under 81% RH (**b**), moisture retention rate under 43% RH (**c**), moisture absorption rate under 10% RH (**d**). Sodium alginate as a positive control. Note: For moisture absorption assay, the samples were oven-dried at 100 °C for 4 h and placed in a saturated K_2_CO_3_ chamber (43% RH) (**a**) or saturated (NH_4_)_2_SO_4_ chamber (81% RH) at 25 °C for the indicated times. The moisture absorption rate (Ra) of a sample was evaluated by the gain of weight. For moisture retention assay, the samples were oven-dried at 100 °C for 4 h and placed in a water chamber to humidify at 25 °C for 36 h and then transferred to a saturated K_2_CO_3_ chamber (43% RH) to dehydrate at 25 °C for 24 h (**c**). The samples were then further dried in a silica gel chamber (10% RH) at 25 °C for the indicated times (**d**). The moisture retention rate (Rr) was evaluated by the weight loss of the sample.

Polysaccharides’ hydroxyl and carboxyl groups can form hydrogen bonds with water molecules, allowing them to bind a large amount of water. Therefore, polysaccharides possess strong hygroscopic and moisturizing activities, making them effective moisturizers that protect the skin from aging [70]. To explore the potential application of water-soluble polysaccharides from *C. aponinum* SCSIO-45682 in cosmetics, the moisture absorption and retention capacities of the crude polysaccharides were determined gravimetrically and compared with sodium alginate. Figure 3 shows that crude polysaccharides exhibited excellent moisturizing activities.

As exhibited in Figure 3a, the moisture absorption rate (Ra) of both crude polysaccharides and sodium alginate at 43% RH slightly increased in 24 h, and the Ra of *C. aponinum* polysaccharides was comparable to that of sodium alginate at each time point (*p* > 0.05). After being exposed to 43% RH for 24 h, the Ra of *C. aponinum* polysaccharides and sodium alginate were 12.81% and 12.08%, respectively. The Ra values of the two samples at 81% RH were comparable with each other (*p* > 0.05) and higher than those at 43% RH. After subjecting to 81% RH for 24 h, the Ra of *C. aponinum* polysaccharides and the positive control reached 24.95% and 23.13%, respectively, which indicates a comparable in vitro hygroscopic property of *C. aponinum* polysaccharides to sodium alginate (Figure 3b). By comparison, Li et al. [14] reported that for moisture absorption ability, after exposing to 43% RH for 24 h, *Nostoc* polysaccharide, chitosan, and urea showed a Ra value of 10.1%, 6.3%, and 5.8%, respectively. Moreover, under the 81% RH condition for 24 h, the Ra values of the above three samples were 15.0%, 10.2%, and 12.7%, respectively. These results are lower than those of *C. aponinum* polysaccharides and sodium alginate in our study.

For the investigation of moisture retention capability, *C. aponinum* polysaccharides and sodium alginate were first placed in a water-humidified chamber to absorb moisture for 36 h until reaching constant weight and then were dehydrated under 43% RH condition. According to Figure 3c, in the first 4 h, water was lost gradually in the two samples and much slower in *C. aponinum* polysaccharides than in sodium alginate (*p* < 0.05). After that, there was no significant difference between the moisture retention rate (Rr) of them (*p* > 0.05), which was 17.33% and 18.16%, respectively, at 24 h under 43% RH condition. The two samples were further subjected to 10% RH for 24 h. Figure 3d depicted that during the first 12 h, the Rr values of *C. aponinum* polysaccharides and sodium alginate at 10% RH had no significant difference (*p* > 0.05). At 24 h, the Rr of the positive control (10.44%) was higher than that of crude polysaccharides from *C. aponinum* (7.70%). Taken together, these results revealed the strong in vitro moisture-retention capacity of *C. aponinum* polysaccharide as compared to sodium alginate.

It is worth noting that the porous and fibrous structure of crude polysaccharides (Figure 1a,b) may help with their moisture retention activity. For example, Li et al. [71] reported that for okara particles, the water-holding capacity was related to the specific surface area and the porous structure. Qiu et al. [72] reported that for rehydrated shiitake mushrooms, the water-holding capacity had a correlation with its cell wall’s fibrous material, such as chitin and beta-glucans, via osmotic bonding.

### 2.9. Cytotoxicity Activity of CAP on LO2 and HepG2

**Figure 4:**
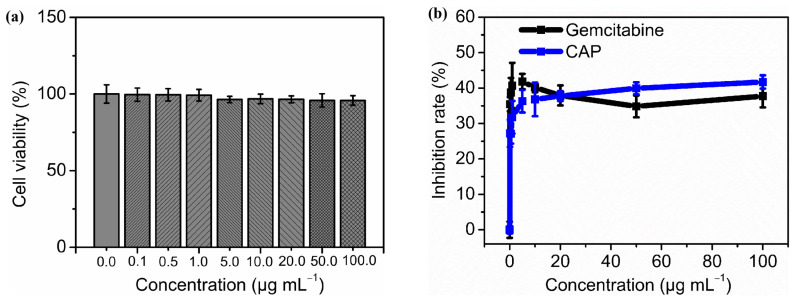
Cytotoxicity activity of CAP on LO2 and HepG2 cells in vitro. The cell viability of LO2 treated with different concentrations of CAP (0.0, 0.1, 0.5, 1.0, 5.0, 10.0, 20.0, 50.0, 100.0 µg mL^−1^) for 48 h (**a**) and the growth inhibition rate of CAP with same concentrations on HepG2 cell after 48 h treatment. Gemcitabine was used as a positive control (**b**).

As illustrated by Figure 4a, after treatment with different concentrations of CAP (0.1, 0.5, 1.0, 5.0, 10.0, 20.0, 50.0, and 100.0 µg mL^−1^) for 48 h, the human fetal hepatocyte cell LO2 showed no decline in viability (*p* > 0.05 compared with negative control). This result showed no hepatotoxicity of CAP on the LO2 cell line were observed among tested concentrations.

Figure 4b reveals the antiproliferative activity of CAP on HepG2 human liver cancer cells. Compared to negative control, all treatment groups with different concentrations of CAP or gemcitabine showed highly significant differences in the inhibition rates (*p* < 0.01 or *p* < 0.001). The inhibitory effect on HepG2 cells of gemcitabine did not demonstrate a clear dose-dependent trend, showing an average inhibition rate of 38.42% (IC_50_ = 20.63 µg mL^−1^). There were no significant differences in the inhibition rates among the groups treated with various concentrations of gemcitabine (*p* > 0.05). By comparison, there were highly significant differences in the inhibition rates among different CAP groups (*p* < 0.001). The lowest inhibition rate of CAP was 27.23%, obtained at 0.1 µg mL^−1^, while the highest effect reached 41.72% at 100.0 µg mL^−1^ (IC_50_ = 93.07 µg mL^−1^).

With regard to some polysaccharides, the antitumor activity was inversely proportional to their relative molecular weight [55,73]. In our study, however, CAP was a high molecular compound (4596.64 kDa) with good HepG2 antiproliferation activity (Table 3, Figure 4). Chen et al. [74] reported that the water-soluble polysaccharides from *Tribonema* sp. with a molecular weight of 197 kDa exhibited a good HepG2 inhibition effect. At a concentration of 50 µg mL^−1^, its inhibition rate was approximately 23%, significantly lower than that of CAP (39.94% at 50 µg mL^−1^). Li et al. [75] found that a low molecular weight water-soluble polysaccharide (18.8 kDa) from *Phellinus linteus* inhibited HepG2 cells by approximately 18% at 50 µg mL^−1^, significantly lower than that of CAP. Fan et al. [76] reported that water-soluble polysaccharides from the economic brown alga *Sargassum fusiforme* (299 kDa) showed a rising inhibition effect on HepG2 with increasing polysaccharide concentration and incubation time. After 48 h of incubation, at the lowest sample concentration of 125 µg mL^−1^, the inhibition rate was only about 4%, while at 1000 µg mL^−1^, it came to around 38%. Additionally, it showed an IC_50_ of 1158.6 µg mL^−1^, significantly higher than CAP (93.07 µg mL^−1^).

Globally, liver cancer is one of the most common cancers and the third leading cause of cancer-related deaths [77]. It is an aggressive malignancy prone to poor prognosis. Current treatments for liver cancer are primarily composed of chemotherapy, interventional therapy, and surgical intervention. However, each therapy has limited clinical benefits and is always accompanied by hepatotoxicity, drug resistance, and other adverse effects [76]. Therefore, finding natural compounds with higher biocompatibility and degradability as alternative therapies has become an emerging research field. In this study, CAP revealed significant growth inhibition activity on HepG2 cells and none hepatotoxicity on LO2 cells, up to the concentration tested. In the future, we will continue to conduct apoptosis detection, relevant cytokine detection, and in vivo antitumor assay to further evaluate the anti-liver cancer effects of CAP and its potential application in pharmaceuticals and functional food.

## 3. Materials and Methods

### 3.1. Cyanobacterial Strain and Cultivation

*C. aponinum* SCSIO-45682 was isolated by streak plate method from water samples collected from Sanya, Hainan province, China (E 109°19′38″, N 18°18′31″). It was identified by morphology and phylogenetic analysis based on 16S ribosomal RNA gene (16S rDNA) and 16S-23S ribosomal DNA internal transcribed spacer (ITS) sequences [33]. This strain was grown in a 2000 mL Erlenmeyer flask containing 1200 mL f/2 medium as a standing culture shaken manually 6 times per day. The f/2 medium was added with 8.4 g L^−1^ NaHCO_3_ for the maximum growth and polysaccharides production of this strain [33]. The medium composition is as follows: 100 mM NaHCO_3_, 4.7 mM NaNO_3_, 0.13 mM NaH_2_PO_4_·2H_2_O, 11.8 µM Na_2_EDTA·2H_2_O, 11.8 µM FeCl_3_·6H_2_O, 0.91 µM MnCl_2_·4H_2_O, 0.08 µM ZnSO_4_·7H_2_O, 0.04 µM CoCl_2_·6H_2_O, 0.04 µM CuSO_4_·5H_2_O, and 0.03 µM Na_2_MoO_4_·2H_2_O in 25‰ natural seawater collected from euphotic layer of the South China Sea. The cultures were incubated at 25 ± 1 °C, 150 ± 5 μmol photons m^−2^ s^−1^ with a 24 h:0 h (light: dark) photoperiod for 14 days. Afterwards, the cells were harvested by centrifugation and then washed three times with deionized water, freeze-dried, and stored at −20 °C before polysaccharide extraction. 

### 3.2. Polysaccharides Preparation from C. aponinum SCSIO-45682

Crude polysaccharides from *C. aponinum* SCSIO-45682 were obtained using the hot water extraction method reported by Khan et al. [78]. Briefly, deionized water was added to *C. aponinum* SCSIO-45682 freeze-dried powder, and the resultant suspension was subject to sonication (160 W, 28 kHz, 25 °C) for 20 min in a water bath. Afterward, the hot water extraction was conducted with stirring in a water bath. The supernatant was combined and concentrated to 1/4 of its original volume using a rotary evaporator. Four-fold absolute ethanol was added subsequently for the precipitation of polysaccharides at 4 °C overnight. The precipitate was harvested by centrifugation at 10,000 rpm for 10 min and redissolved in a small volume of deionized water. 0.05% (*w:v*) of papain was added to the solution for the removal of proteins. 10% (*v:v*) of 30% (*w:v*) H_2_O_2_ was then used to remove pigments from the crude polysaccharide extract. After boiling for 10 min, the extract was collected by centrifugation at 10,000 rpm for 10 min, and subsequently dialyzed against deionized water for 48 h (500 Da cutoff), and freeze-dried at −50 °C for 48 h to obtain water-soluble crude polysaccharides. The extraction yield (Y) was calculated based on the following formula:

Y (%, w:w) = weight of polysaccharides/weight of *C. aponinum* SCSIO-45682 (1)

The hot water extraction was preliminarily tested regarding the following four independent variables, i.e., solid-liquid ratio (A), extraction temperature (B), extraction time (C), and extraction times (D). Based on that, an orthogonal assay with four factors at three levels L_9_ (3^4^) was carried out to optimize the crude polysaccharide yield.

For further purification, freeze-dried crude polysaccharides were re-dissolved in ultrapure water in a final concentration of 10 mg mL^−1^ and then applied to a cellulose DEAE-52 column (3.5 cm × 40 cm) equilibrated with water. The column was eluted with water, followed by a series of NaCl solutions (0.1, 0.3, 0.4, 0.5, 0.8, 1.0 M) at a flow rate of 2 mL min^−1^. The total carbohydrate concentration from each elute was monitored by the phenol-sulfuric acid method [79]. Elutes from the predominantly major peak were combined, dialyzed, and lyophilized. The isolated polysaccharide fraction was further purified by a Sephadex G-100 column (2.5 cm × 30 cm) by elution using ultrapure water at 1.5 mL min^−1^. The total carbohydrate concentration from each elute sample was monitored by the phenol-sulfuric acid method [79]. In the final, a homogeneous fraction obtained (*Cyanobacterium aponinum* polysaccharide, CAP) was lyophilized and used for the following experiments. 

### 3.3. SEM

The surface morphology of crude polysaccharides was observed by scanning electron microscope (SEM) (Sigma 300/VP, Zeiss, Oberkochen, Germany). The freeze-dried sample was adhered to specimen holders with double-sided carbon tape, coated with a thin gold layer, and analyzed in a high-vacuum chamber at 3.0 kV and under 50,000× magnification [80].

### 3.4. AFM

The ultrastructural features of crude polysaccharides were determined by atomic force microscopy (AFM) (Bruker Dimension ICON, Karlsruhe, Germany), based on the method reported by [81]. The sample was dissolved in distilled water at a concentration of 1 μg mL^−1^, subject to ultrasonic treatment for 30 min, and then 3 µL sample solution was dispersed on cleaved micas, which was subsequently vacuum-dried at 120 °C for 0.5 min before analysis.

### 3.5. Distribution of Molecular Weight

The homogeneity and molecular weight (Mw) distribution of CAP were measured by size exclusion liquid chromatography based on the previous method [80]. In brief, CAP dissolved in 0.1 M NaNO_3_ was injected into HPLC equipped with three tandem columns (Shodex OH-pak SB-805, 804, and 803, 300 × 8 mm, Showa Denko K.K., Tokyo, Japan). The column temperature was held at 45 °C. 0.1 M NaNO_3_ served as the mobile phase, which eluted for 100 min with a flow rate of 0.4 mL min^−1^. A DAWN HELEOS-II laser photometer detector (He-Ne laser, λ = 663.7 nm, Wyatt Technology Co., Santa Barbara, CA, USA) was used to measure the weight and number-average molecular weight (Mw and Mn) and polydispersity index (Mw/Mn). A differential refractive index detector (Optilab T-rEX, Wyatt Technology Co., Santa Barbara, USA) was simultaneously connected to give the concentration of fractions and the refractive index increment (dn/dc) value.

### 3.6. Monosaccharide Composition Determination

The monosaccharide composition of CAP treated with 2 M trifluoroacetic acid was determined by high-performance liquid chromatography (HPLC, ICS5000, Thermo Fisher Scientific, Waltham, MA, USA) equipped with an anion-exchange column (Dionex™ CarboPac™ PA20, 150 × 3 mm) and a pulsed amperometric detector (Dionex ICS 5000 system, Sunnyvale, CA, USA). The column temperature was 30 °C. Mobile phase A was 0.1 M NaOH, and mobile phase B was composed of 0.1 M NaOH and 0.2 M NaAC. The gradient program was 95:5 (*v:v*) at 0 min, 80:20 (*v:v*) at 30 min, 60:40 (*v:v*) at 30.1 min, 60:40 (*v:v*) at 45 min, 95:5 (*v:v*) at 45.1 min, 95:5 (*v:v*) at 60 min at 0.5 mL min^−1^ [80].

### 3.7. Chemical Composition Measurement

CAP was freeze-dried and weighed carefully. The total carbohydrate content of CAP was measured by the phenol-sulfuric acid method with D-glucose as a standard [79]. The protein content of CAP was measured by bicinchoninic acid assay with bovine serum albumin as a standard [82]. The sulfate content of CAP was determined by barium chloride-gelatin turbidity method, using potassium sulfate as a standard [83]. The uronic acid content of CAP was measured by meta-hydroxydiphenyl assay using D-glucuronic acid as a standard [84]. The total phenolic compound content of CAP was determined by using a Folin-Ciocalteu assay with gallic acid as a standard [85]. The total carbon content of CAP was determined using an organic elemental analyzer (Elementar model Vario EL III) in the CHNS mode [86].

### 3.8. Fourier-Transform Infrared Analysis

The functional groups in CAP were determined by a Fourier-transform infrared (FT-IR) system (IR Affinity-1, Shimadzu, Tokyo, Japan) within the region of 4000–500 cm^−1^ at a resolution ratio of 1 cm^−1^.

### 3.9. Moisture Absorption and Retention Activity of Crude Polysaccharides

The moisture absorption and retention assay of crude polysaccharides was performed using the method established by Li et al. [14] with some modifications: for the evaluation of moisture retention capacity, the samples were first placed in a humidification chamber containing deionized water for 36 h until reaching constant weight and then transferred to a humidity chamber containing saturated K_2_CO_3_ (43% relative humidity, RH) for 24 h. The samples were finally transferred to a desiccation chamber containing dried silica gel (10% RH) for another 24 h.

### 3.10. Cytotoxicity Activity of CAP on LO2 and HepG2 Cell Lines

The cytotoxicity of CAP on human fetal hepatocyte cell line LO2 (purchased from iCell Bioscience Inc., Shanghai, China) was evaluated by MTT (3-(4,5-dimethylthiazol-2-yl)-2,5-diphenyltetrazolium bromide) assay. Briefly, LO2 cells were cultured in MEM medium containing 10% fetal bovine serum, 100 U mL^−1^ penicillin, and 100 μg mL^−1^ streptomycin at 37 °C with 5% CO_2_. The cells were seeded in a 96-well plate at a concentration of 1 × 10^4^ cells mL^−1^, cultivated under normal growth conditions overnight, and then incubated for 48 h with different concentrations of CAP (0.0, 0.1, 0.5, 1.0, 5.0, 10.0, 20.0, 50.0, and 100.0 µg mL^−1^) of 100 µL. Subsequently, CAP was removed and replaced by media with 100 µL of 0.5 mg mL^−1^ MTT, and then the cells were incubated for another 4 h. Finally, 100 µL of dimethyl sulfoxide (DMSO) was added to stop the reaction. The absorbance at 570 nm was determined by an automatic Elisa analyzer (Epoch2, Biotech, Boston, MA, USA) after gently shaking for 10 min. The cytotoxicity of CAP on LO2 cells was represented as the cell viability based on the following formula:

Cell viability (%) = A_sample_/A_control_ × 100 (2)

where A_sample_ represents the absorbance of the treatment group, whereas A_control_ represents the absorbance of the negative control group, which contains all the reagents except for CAP.

The growth inhibitory activity of CAP on human hepatocellular carcinoma cell line HepG2 (purchased from iCell Bioscience Inc.) was conducted under the same conditions mentioned above. Gemcitabine was used as a positive control. The inhibition rate on the growth of HepG2 was calculated based on the formula below:

Inhibition rate (%) = 1 − A_sample_/A_control_ × 100 (3)

where A_sample_ represents the absorbance of the treatment group, whereas A_control_ represents the absorbance of the negative control group, which contains all the reagents except for CAP or gemcitabine.

### 3.11. Statistical Analysis

Statistical analysis was performed using a one-way analysis of variance (ANOVA) with subsequent post hoc multiple-comparison Tukey tests using Origin Pro 8.5 software (OriginLab Corporation, Northampton, MA, USA). The data were presented as mean ± standard deviation of three biological or technical replicates. The value of *p* < 0.05 was considered to be statistically significant.

## 4. Conclusions

In this research, the extraction optimization, isolation, structural characterization, and biological activity evaluation of water-soluble polysaccharides from *C. aponinum* SCSIO-45682 were systematically studied. The crude water-soluble polysaccharides reached an extraction yield of 17.02% DW and showed a porous, fibrous structure, which was in line with its notable moisture absorption and retention capacities comparable to sodium alginate. One homogeneous component (CAP) was characterized as a high molecular weight acidic heteropolysaccharide rich in fucose. CAP showed a significant growth inhibition effect on hepatocellular carcinoma HepG2 cells with none hepatotoxicity on LO2 cell within the tested concentration. Taken together, our findings indicated the potential of water-soluble polysaccharides from *C. aponinum* SCSIO-45682 as a possible moisturizer and anticancer addictive applied in cosmetical and pharmaceutical industries, but further research is needed about its in vivo antitumor activity and related molecular mechanisms.

## Figures and Tables

**Table 1:** Intuitive analysis of the orthogonal assay results.

No.	Levels of Factor				Crude Polysaccharide Yield (% DW)
	A	B	C	D	
1	1 (1:30)	1 (60 °C)	1 (1 h)	1 (3 times)	8.37
2	1	2 (70 °C)	2 (2 h)	2 (4 times)	13.01
3	1	3 (80 °C)	3 (3 h)	3 (5 times)	17.02
4	2 (1:40)	1	2	3	15.85
5	2	2	3	1	13.79
6	2	3	1	2	11.55
7	3 (1:50)	1	3	2	15.80
8	3	2	1	3	11.60
9	3	3	2	1	11.82
K1	12.80	13.34	10.51	11.33	
K2	13.73	12.80	13.56	13.45	
K3	13.07	13.46	15.54	14.82	
R	0.93	0.66	5.03	3.50	

Note: Ki is obtained by adding any number of columns corresponding to the i factor. R is the difference between the maximum value and the minimum value of Ki of any column.

**Table 2:** Analysis of variance of the orthogonal assay results.

Source of Variation	Sum of Squares	Degree of Freedom	Mean Square	*F* Value	Significance
A	0.10	2	0.05	1.99	
C	2.81	2	1.41	58.08	*
D	1.36	2	0.68	28.12	*
B	0.05	2	0.02	/	/

Note: * denotes *p* < 0.05, representing a statistical difference of this factor among other factors.

**Table 3:** Molecular weight, chemical composition, and monosaccharide composition of CAP.

Index	Values
Molecular weight (kDa)	4596.64
Chemical composition (% CAP)	
Total carbon	34.68 ± 0.01
Total carbohydrate	34.69 ± 1.05
Protein	0.84 ± 0.03
Sulfate	18.06 ± 0.05
Uronic acid	12.96 ± 0.52
Total phenolic compound	0.42 ± 0.29
Monosaccharide composition (%, molar ratio)	
L-Fucose	15.27
L-Rhamnose	4.53
L-Arabinose	0.31
D-Galactose	11.39
D-Glucose	7.08
D-Xylose	0.26
D-Mannose	4.07
D-Ribose	4.43
D-Galacturonic acid	8.64
D-Glucuronic acid	1.30

## Data Availability

The original contributions presented in the study are included in the article/Supplementary Material; further inquiries can be directed to the corresponding author/s.

## Supplementary Materials

The following supporting information can be downloaded at https://www.mdpi.com/article/10.3390/molecules29194556/s1, Figure S1: Effects of solid-liquid ratio (a), extraction temperature (b), extraction period (c), and extraction times (d) on the yield of crude water-soluble polysaccharides from *C. aponinum* SCSIO-45682; Figure S2: Molecular weight distribution of CAP. Refs. [52,87,88] are cited in Supplementary Material.
