# Supplementary material for: Isolation, Characterization, Moisturization and Anti-HepG2 Cell Activities of a Novel Polysaccharide from Cyanobacterium aponinum
Source: Molecules. 2024 Sep 25;29(19):4556. doi: 10.3390/molecules29194556 (PMC11478272; doi:10.3390/molecules29194556)
Supplement: Supplementary file 1 [file molecules-29-04556-s001.zip › molecules-3144774-supplementary.pdf]

Isolation, Characterization, Moisturization and Anti-HepG2 cell Activities of a Novel Polysaccharide from *Cyanobacterium aponinum*

Zishuo Chen [1,2,†], Jiayi Wu [1,3,†], Na Wang [1,2,4,†], Tao Li [1], Houbo Wu [1], Hualian Wu [1,*] and Wenzhou Xiang [1,*]

[1] CAS Key Laboratory of Tropical Marine Bio-Resources and Ecology, Guangdong Key Laboratory of Marine Materia Medica, Institution of South China Sea Ecology and Environmental Engineering, RNAM Center for Marine Microbiology, South China Sea Institute of Oceanology, Chinese Academy of Sciences, Guangzhou 510301, China; zschen1030@gmail.com (Z.C.); wujiayi@ipm-gba.org.cn (J.W.); wangna@usc.edu.cn (N.W.); taoli@scsio.ac.cn (T.L.); wuhoubo@scsio.ac.cn (H.W.)
[2] University of Chinese Academy of Sciences, Beijing 100049, China
[3] Greater Bay Area Institute of Precision Medicine (Guangzhou), Guangzhou 511466, China
[4] School of Basic Medical Sciences, Heyang Medical School, University of South China, Hengyang 421001, China
* Correspondence: hlwu@scsio.ac.cn (H.W.); xwz@scsio.ac.cn (W.X.); Tel.: +86-020-8902-3013 (H.W.); +86-020-8902-3223 (W.X.)
† These authors contributed equally to this work.

Supplementary materials

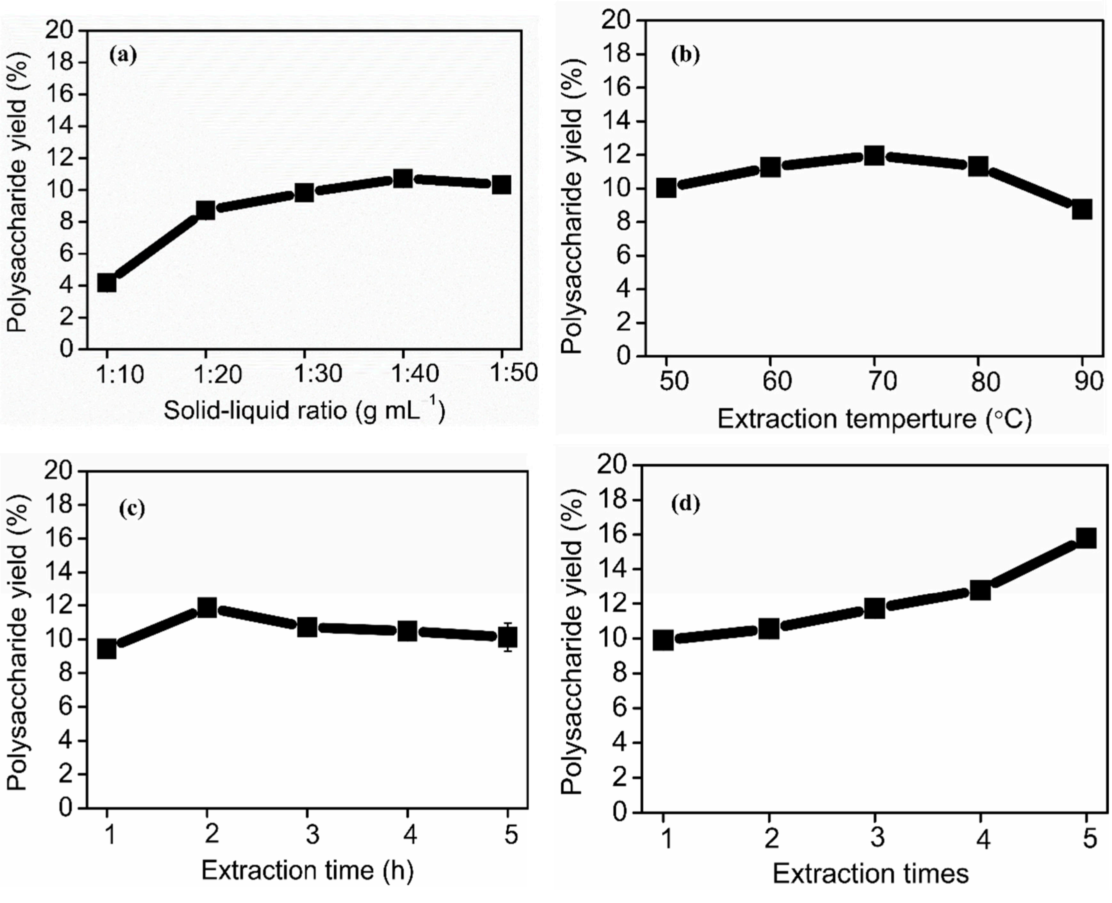

Figure S1. Effects of solid-liquid ratio (a), extraction temperature (b), extraction time (c), and extraction

times (d) on the yield of crude water-soluble polysaccharides from *C. aponinum* SCSIO-45682.

A single-factor experiment about hot water extraction was carried out before an orthogonal assay. When measuring the effects of the solid-liquid ratio on the yield of crude polysaccharides, the extraction temperature was fixed at 80 °C, the extraction time was set to 2 h, and the extraction times was fixed at 3 times. When the effects of extraction temperature were determined, the solid-liquid ratio was set as 1:40 g mL^{-1}, the extraction time was fixed at 2 h, and the extraction times was set at 3 times. When the effects of extraction time were estimated, the solid-liquid ratio, the extraction temperature, and the extraction times were fixed at 1:40 g mL^{-1}, 80 °C, and 3 times, respectively. When the effects of different extraction times were measured, the solid-liquid ratio, the extraction temperature and the extraction time were fixed at 1:40 g mL^{-1}, 80 °C, and 2 h, respectively. Results were shown as a proportion of the crude polysaccharide dry weight of the *C. aponinum* SCSIO-45682 biomass dry weight.

Supplementary results:

Figure S1a depicts that a higher solid-liquid ratio enhances the polysaccharide extraction efficiency via creating a concentration difference between the interior substances within cells and the exterior solvent, which in turn favors mass transfer. Too much liquid, however, would not change much of the driving force as the limitation to mass transfer is more confined to the solid interior [87].

Under hydrothermal conditions, cellulose lattice structure within the cell could be broken down, followed by an increasing solubility of polysaccharides in water. Thus, the extraction yield of polysaccharides increased with the rise of extraction temperature. However, too high temperature might cause the degradation of polysaccharides (Figure S1b). This is in line with the findings of Liu et al. [52].

Time is another important factor affecting extraction yield of polysaccharide. Different extraction period would influence the solvent and solid matrix contact. However, when the cyanobacteria cells ruptured, various compounds such as insoluble substances were also suspended in the solution, resulting in the lower permeability of the solvent [87]. Hence, the yield of crude polysaccharides showed an enhancement followed by a decrease when the extraction time extended (Figure S1c).

According to Figure S1d, the polysaccharide yield increased when the cyanobacterial biomass was extracted for more time. This was consistent with the results of Samavati et al. [88]. Moreover, the extraction effect declined when 6 or 7 times of extraction was conducted in our research (data not shown).

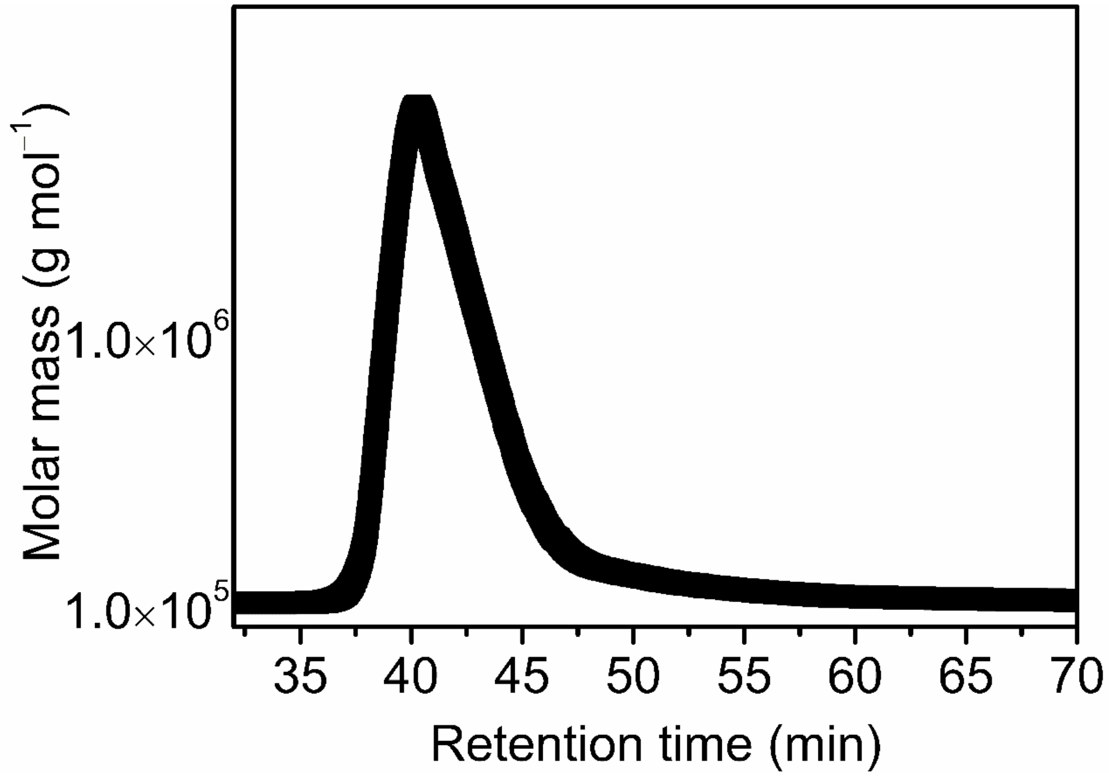

Figure S2. Molecular weight distribution of CAP.

The homogeneity and molecular weight distribution of CAP were determined by high-performance size-exclusion chromatography-multi angle laser light scattering-refractive index detector (HPSEC-MALLS-RI). The result was represented as the profile of the multi angle laser light scattering detector. Data were acquired and processed using ASTRA6.1 software (Wyatt Technology, CA, USA).

References

52. Liu, F.; Chen, H.; Qin, L.; Al-Haimi, A.A.N.M.; Xu, J.; Zhou, W.; Zhu, S.; Wang, Z. Effect and characterization of polysaccharides extracted from *Chlorella* sp. by hot-water and alkali extraction methods. *Algal Res.* **2023**, *70*, 102970.
87. Maran, J.P.; Mekala, V.; Manikandan, S. Modeling and optimization of ultrasound-assisted extraction of polysaccharide from *Cucurbita moschata*. *Carbohydr. Polym.* **2013**, *92*, 2018–2026.
88. Samavati, V.; Manoochehrizade, A. Polysaccharide extraction from *Malva sylvestris* and its anti-oxidant activity. *Int. J. Biol. Macromol.* **2013**, *60*, 427–436.
